# Psychogenic non-epileptic seizures in early Huntington's disease

**DOI:** 10.1136/practneurol-2016-001423

**Published:** 2016-06-21

**Authors:** Filipe Brogueira Rodrigues, Edward J Wild

**Affiliations:** Huntington's Disease Centre, Institute of Neurology, University College London, London, UK

**Keywords:** Huntington disease, pseudoseizures, case reports

## Abstract

Huntington's disease (HD) is a neurodegenerative condition characterised by motor dysfunction with involuntary movements and loss of voluntary control, cognitive deterioration and psychiatric problems. We report a 51-year-old man with early HD who experienced stereotyped episodes of repetitive, purposeless complex movements and unresponsiveness. His neurological examination was compatible with HD as were all investigations. We diagnosed psychogenic non-epileptic seizures. While seizures are common in juvenile-onset HD, they are no more prevalent in adult-onset HD than in the general population. However, neuropsychiatric symptoms are common in HD and can involve a number of different complaints. Patients may experience dissociative attacks soon after manifesting a HD diagnosis. Such episodes can be managed with patient and carer education, cognitive behavioural therapy and anxiolytic selective serotonin reuptake inhibitors.

## Case report

A 51-year-old man with early Huntington's disease (HD) was reviewed at our multidisciplinary HD clinic after developing stereotyped paroxysmal episodes of repetitive, purposeless complex movements and unresponsiveness.

His mother and two brothers had HD. He first attended our clinic at 51 years of age after developing involuntary movements and difficulty with complex tasks at work. We confirmed the clinical diagnosis of early HD after a positive result from *HTT* genetic testing. After this initial appointment, he had informed his employer about the diagnosis, who offered him early retirement on medical grounds—an event perceived by the patient as a major emotionally stressful life event.

A few months later, he started developing stereotyped episodes of repetitive, purposeless complex movements and unresponsiveness followed by transient disorientation, emotional lability and fatigue. His wife videotaped one episode (see online supplementary video 1a). The episodes were occurring about once weekly and this frequency was stable over time. There were no triggers or prodromal symptoms, and each episode lasted approximately 5 min. There was no prior history of mental illness or trauma. The episodes usually started with him staring at or examining an object—such as a spoon or a plastic box. After 30–60 s, repetitive purposeless complex hand and upper limb movements ensued. These were more complex than the mild, fidgety movements of his HD-related chorea. During the episodes, he was awake but unresponsive to verbal stimuli. At the end of the episode, the movements waned and he started to answer to simple commands. For about 10 min afterwards, he was disorientated to place and person with emotional lability and tiredness. He made a complete recovery afterwards.

10.1136/practneurol-2016-001423.supp1 Supplementary video

The patient consulted his general practitioner who referred him to us concerning that the attacks may be epileptic seizures. Two months after the first attack, we reviewed him; his neurological examination between attacks was compatible with HD (see online supplementary video 1b) with no unexpected focal signs. Routine and sleep-deprived electroencephalograms (EEGs) and MR neuroimaging were normal.

We diagnosed probable psychogenic non-epileptic seizures according to the International League Against Epilepsy (ILAE) criteria^1^ on the basis of the presence of predisposing factors, compatible semiology assessed by an experienced neurologist in a video recording, exclusion of an explanatory medical cause and negative investigations, including interictal EEG. We informed him of the diagnosis and the risk factors that had contributed to its development, counselled on de-escalation techniques and gave citalopram for low mood and anxiety. The episodes ceased and 24 months later, he remains event-free.

## Discussion

HD is a hereditary neurodegenerative condition caused by a cytosine–adenine–guanine (CAG) repeat expansion in the *HTT* gene on the short arm of chromosome 4, with consequent production and accumulation of mutant huntingtin protein, which causes neuronal dysfunction and death.^2^ It is clinically characterised by a triad of motor dysfunction with involuntary movements and loss of voluntary control, cognitive deterioration and psychiatric problems.^2^

Seizures are common in juvenile-onset HD^2^ but are no more prevalent in adult-onset HD than in the general population. However, neuropsychiatric symptoms are common in early HD and can involve several different complaints,^2^ including dissociative phenomena.

In our experience, many patients experience functional neurological symptoms soon after manifesting a HD diagnosis—most likely because of increased disease-related anxiety and the inevitable severe stress that accompanies such a diagnosis. In the general population, psychogenic non-epileptic seizures and psychogenic movement disorders are among the most frequent psychologically-mediated syndromes seen by neurologists,^3^ and this is also our experience in the HD clinic. It is often difficult to distinguish between psychogenic movement disorders and psychogenic non-epileptic seizures, since both share causative factors and clinical features; some authors classify both conditions under the same spectrum.^3^ In this particular case, the altered higher mental function and the complexity of the movements made psychogenic non-epileptic seizures more likely.

Psychogenic non-epileptic seizures or dissociative seizures occur in 10–20% of people diagnosed with epilepsy.^4^ They can be clinically similar to any kind of epileptic seizure but are of psychological origin rather than being caused by hypersynchronous discharges in the brain. Attacks that resemble complex partial seizures are a relatively rare manifestation of psychogenic non-epileptic seizures but, in our experience, patients with HD can report any kind of epilepsy-like attack. It remains to be seen whether complex partial seizure-like attacks are more common in this population. Psychogenic non-epileptic seizures are considered to be an abnormal coping mechanism in the spectrum of dissociation. Indeed, patients with this condition have stronger connectivity between brain areas associated with emotion (insula), executive (inferior frontal gyrus and parietal cortex) and movement control (precentral sulcus),^5^ a pattern also observed in conversion disorders.

Although psychogenic non-epileptic seizures are most frequent in women in their 20s and 30s, they can occur in anyone.^1^ There is a previous history of psychological trauma or psychiatric co-morbidities in approximately 70% of patients.^1^ Up to half of patients have a precipitating life event.^1^ The semiology of psychogenic non-epileptic seizures can resemble most types of epileptic seizures but some aspects may increase the clinical suspicion: triggering by psychological stress, long duration, fluctuating course, asynchronous movements, pelvic thrusting, forced eye closure, ictal crying, memory recall and signs of emotional distress.^1^ ^6^

The diagnostic gold standard is video EEG, with the absence of ictal electroencephalographic abnormalities before, during and after the attack.^1^ The use of this technique is obligatory to make a *documented diagnosis* based on the ILAE criteria.^1^ Nevertheless, it is possible to establish the diagnosis with a lower degree of certainty. A clinically established diagnosis should be made when a conventional ictal EEG detects no abnormalities and the semiology is compatible with psychogenic non-epileptic seizures; a normal interictal EEG and a clinically witnessed event or clinically reviewed video-recording of an event suffice to allow a *probable diagnosis*; and finally, at the lower end of diagnostic certainty, a *possible diagnosis* needs a normal interictal EEG and a witness or self-reported description of a compatible event.^1^

These non-epileptic attacks can have adverse quality-of-life consequences and a high rate of recurrence.^7^ Thankfully, early and accurate recognition of psychogenic non-epileptic seizures improves the prognosis.^8^ The treatment plan should encompass discussing and explaining the diagnosis to the patient and carers, withdrawal of antiepileptic drugs with special attention to discontinuation syndromes, and psychiatric and/or psychological interventions.^6^

**Key points**

- Huntington's disease (HD) is a hereditary neurodegenerative condition caused by a cytosine–adenine–guanine repeat expansion, characterised by motor dysfunction, cognitive deterioration and psychiatric problems.
- Epileptic seizures are a common feature of juvenile-onset HD.
- Seizures are no more prevalent in adult-onset HD than in the general population, but anxiety phenomena are very common in HD and can produce dissociative phenomena that clinically mimic epileptic events.
- Many patients experience functional neurological symptoms soon after manifesting a HD diagnosis.
- Non-epileptic seizures should be managed with patient and carer education, withdrawal from antiepileptic drugs, and psychiatric and/or psychological interventions.
